# Transcriptome analysis reveals the accelerated expression of genes related to photosynthesis and chlorophyll biosynthesis contribution to shade-tolerant in *Phoebe bournei*

**DOI:** 10.1186/s12870-022-03657-y

**Published:** 2022-06-01

**Authors:** Jing An, Xiaoli Wei, Honghao Huo

**Affiliations:** 1 College of Forestry, Guizhou University, Guiyang, 550025 China; 2 Geography & Environmental Science College, Guizhou Normal University, Guiyang, 550025 China; 3 Institute for Forest Resources and Environment of Guizhou, Guizhou University, Guiyang, 550025 China

**Keywords:** *Phoebe bournei*, Chlorophyll, Photosynthesis, Transcriptome

## Abstract

**Background:**

Phoebe bournei (*P. bournei*) is an important and endemic wood species in China. However, the plantation, nursing, and preservation of *P. bournei* are often affected by light. To investigate its physiological changes and molecular mechanism of low light tolerance, two-year-old *P. bournei* seedlings were subjected to different shading conditions. With the increase of light intensity in the shade, the leaf color of *P. bournei* seedlings became darkened, the aboveground/underground biomass significantly increased, the content of chlorophyll increased and the net photosynthetic rate significantly increased.

**Results:**

de novo transcriptome analysis showed that 724 and 3,248 genes were differentially expressed due to low light intensity at T1 (35% light exposure) and T2 (10% light exposure), respectively, when compared to the controls. Furthermore, the differentially expressed genes (DEGs) were implicated in photosynthesis, nitrogen metabolism, plant hormone signal transduction, biosynthesis of secondary metabolites, and protein processing in the endoplasmic reticulum by functional enrichment analysis. Moreover, the expression of *HSP*, *CAB*, *HEMA1*, *GSA*, *DVR*, *MYB*, *bHLH*, *PORA*, *CAO*, *GLK*, and photosystem I and II complex-related genes significantly increased after low light exposure at T2 and T1.

**Conclusions:**

The present study suggests that the rapid growth of *P. bournei* seedlings under shading conditions may be the result of the accelerated expression of genes related to photosynthesis and chlorophyll biosynthesis, which enable plants to maintain a high photosynthesis rate even under low light conditions.

**Supplementary Information:**

The online version contains supplementary material available at 10.1186/s12870-022-03657-y.

## Background

Light is an important ecological factor that affects plant growth and plays an important role in forest regeneration. It takes a long way for different plants in forests, especially shade-tolerant woody plants, to go from seedlings to adult trees. Shade-tolerant tree species can tolerate varying degrees of low light environment during the seedling stage, and grow well under suitable low-light conditions [1, 2]. Different shade-tolerant plant species showed different degrees of shade tolerance, inducing the reconstruction of morphology, growth and habits [3, 4]. These changes also occurred in leaf morphology, biomass ratio, photosynthetic physiological characteristics, and so on [1, 2]. In general, as the light intensity increased, the chlorophyll content of the leaves of shade-tolerant plants decreased, while the chlorophyll a/b content decreased as the light intensity increases [5]. In addition, the carotene content of shade-tolerant plants increased with the shade [6]. Simultaneously, the above-ground biomass increases corresponding to the underground biomass [7]. In addition, shade-tolerant plants have higher photosynthetic physiological characteristics such as the maximum net light rate and light saturation point than shade-intolerant plants. In recent years, with the continuous improvement of cultivation methods, people in some areas often shade plants in certain seasons to obtain better shade-tolerant plant growth [8].

A significant number of studies has revealed numerous genes related to shade tolerance. The downstream genes required for the integration between phytochromes, PhyA and PhyB, and light and hormone signaling pathways were considered to be involved in different plant tolerance responses to shadows [9–11]. Although the research on plant tolerance to negative has achieved great results [12], the underlying mechanisms remain quite complex. RNA sequencing (RNA-seq) is a promising tool to investigate the gene transcript profiling under different conditions in plants [13–15].

*Phoebe bournei* (Hemsl.) is a rare tree species with special national secondary protection in China, mainly distributed in southeast Asia, and southeast and southwest of China. *P. bournei*, a shade-tolerant species, is a unique precious timber and ornamental forest tree species in China, and the growth of its seedlings accelerates under dark conditions [16]. The use of artificial shading nets and suitable understory lighting conditions resulted in the elevated afforestation efficiency of *P. bournei* and higher yield potentials of other crops [17–19]. In the present study, a comparative transcriptomic approach was used to investigate the transcriptome differences among different shading conditions to identify the genes and regulatory mechanisms associated with low-light tolerance in *P. bournei*, considering that physiological functions mainly reflect the changes of photosynthetic physiology at the transcription level. In this process, the transcriptome of *P. bournei* in 100% light exposure was compared with 35% and 10% light exposure, respectively. Briefly, the present study aimed to investigate the effects of low-light intensity on *P. bournei* and the mechanism governing the shading tolerance of *P. bournei*. The present study is the first to report on the molecular mechanism of shading tolerance in *P. bournei*.

## Results

### Phenotypic characterization and physiological changes of *P. bournei* seedlings under different light treatments

**Fig. 1:**
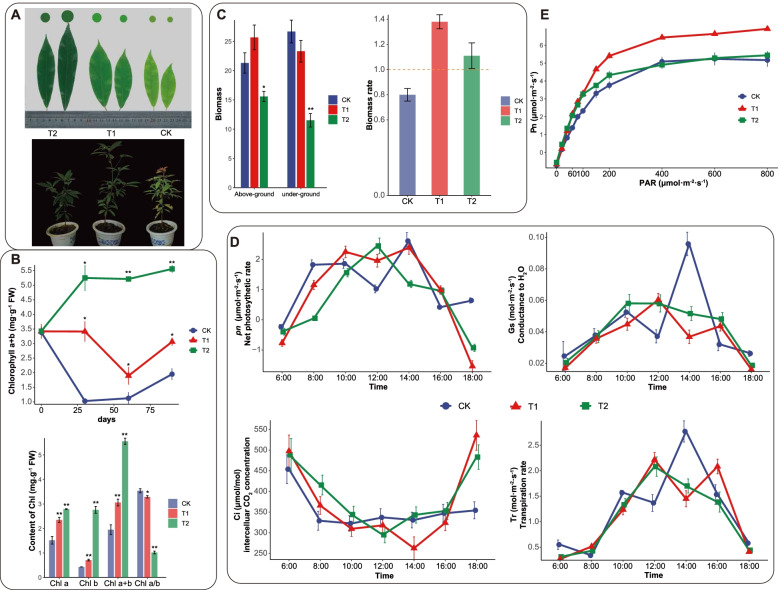
Phenotypic characterization and physiological changes of *P. bournei* under different shading treatments: CK represent the 100% light supply, T1 represents the 35% light, and T2 represents the 10% light. **A** Morphological comparisons under distinct treatments after 30 days. Under 10% illumination, the leaves of *P. bournei* had the deepest color and the largest shape. Under 100% illumination, the leaves were light green and the shapes were small. **B** The chl content comparisons among the different treatments after 30 days is shown. As the degree of shading increased, the content of chlorophyll, especially the content of chlorophyll b, significantly increased, while the ratio of chlorophyll a/b decreased. (T-test, * *P* < 0.05; ** *P* < 0.01). **C** Changes in the biomass of *P. bournei* aboveground and underground under different shading treatments. (T-test, * *P* < 0.05; ** *P* < 0.01) **D** Changes in the *P. bournei* net photosynthetic rate, stomatal conductance, intercellular CO_2_ concentration, and transpiration rate under different shading treatments. **E** The curve for the net photosynthetic rate with light intensity

Through the observation of the leaf area, chlorophyll content and other photosynthetic pigments, biomass, photosynthetic diurnal changes, and light response parameters of *P. bournei* seedlings, the effects of different shading treatments on the growth of *P. bournei* seedlings were comprehensively evaluated. The color of the leaves continued to dark with the decrease of the light intensity after 30 days of shading treatment (Fig. 1A). The chlorophyll content of *P. bournei* after 30, 60 and 90 days of shading treatment was measured. The results showed that the chlorophyll content changes of *P. bournei* seedlings were relatively stable after 30 days of shading treatment (Fig. 1B). At the same time, the composition of chlorophyll content under three light intensities was determined at 30 days. The chlorophyll a content for T1 (35% light intensity) and T2 (10% light intensity) were significantly higher than that for CK (100% light intensity) (Fig. 1B, *P* < 0.001). The chlorophyll b content for T2 and T1 was also significantly higher than that for CK (*P* < 0.05). Compared with CK, the content of chlorophyll a + b for T2 (*P* < 0.001) and T1 (*P* < 0.01) significantly increased. The chlorophyll a/b ratio for T2 significantly decreased (Fig. 1B, *P* < 0.01). Under low light conditions, *P. bournei* increased the content of chlorophyll b and reduced the ratio of chlorophyll a/b to capture solar energy on demand under low light conditions, maintaining the photosynthetic process (Fig. 1B). Compared to CK, the chlorophyll a/b ratio for T1 did not significantly decrease (*P* = 0.22). The aboveground/underground biomass increased, with the CK as the smallest change among the three treatments. Furthermore, the biomass of T1 and T2 increased by 38.8% and 72.5% compared with the control, respectively (*P* < 0.05, Fig. 1C). The overall trend of the daily changes in the net photosynthetic rate of *P. bournei* was T1 > T2 > CK, while the stomatal conductance (G_s_) and transpiration rate (T_r_) for the T1 and T2 changes was smooth relative to CK. The values of Tr and G_s_ were both low in the morning and evening and high at noon; while the Ci was opposite to the trend line of P_n_. Furthermore, the trend of the CO_2_ concentration were the same between different light intensities (Fig. 1D). The trend of Pn showed that *P. bournei* severely stressed at 100% light intensity. The light saturation point (LSP) and maximum photosynthetic rate (P_max_) were all the highest in T1, followed by CK and T2, and the parameters of T1 was 23.56% and 24.21% higher than CK, respectively. The light compensation point (LCP) and respiration rate in dark (R_d_) decreased with the low light intensity (Table 1). There was no significant difference in various indicators between CK and T2 (Fig. 1E).

**Table 1:** Differences in photosynthetic traits in *P. bournei* at the different light intensity

Light intensity	LSP (μmol·m^−2^·s^−1^)	*P *_max_ (μmol·m^−2^·s^−1^)	LCP (μmol·m^−2^·s^−1^)	*R*d (μmol·m^−2^·s^−1^)
CK (100%)	488.83 ± 6.253^b^	5.41 ± 0.382^b^	14.20 ± 0.404^a^	0.71 ± 0.02^a^
T1(35%)	604.17 ± 18.406^a^	6.72 ± 0.345^a^	13.00 ± 0.126^a^	0.54 ± 0.035^b^
T2 (10%)	501 ± 19.655^b^	5.32 ± 0.222^b^	9.48 ± 0.691^b^	0.50 ± 0.067^b^

Values were means ± SE (*n* = 3). Analysis of difference based on t tests of independent samples

^a^significant difference in the same column, i.e., *p*_ value < 0.05, ^b^no significant

### Gene differential expression analysis and function classification of DEGs

We performed deep transcriptome analysis using RNA-seq after 30 days of treatment to understand the mechanisms of the low-light stress of *P. bournei*. A total of 3,248 DEGs were identified between T2 and CK. Among these 3,248 DEGs, 1,273 and 1,975 genes were upregulated and downregulated, respectively (Fig. S1A). Furthermore, 191 genes were upregulated and 398 genes were downregulated between T1 vs CK. Similarly, between T1 vs T2, 267 genes were upregulated and 358 genes were downregulated (Fig. S1A). In the Venn graph, 589 differential genes were expressed in CK and T1. Among the shared differential genes, 27 genes were downregulated and one gene was upregulated. Furthermore, 3,248 differential genes were expressed in CK and T2. Among these, 205 genes were downregulated and 98 genes were upregulated. Similarly, 625 differential genes were expressed in T1 and T2. Among these, 176 genes were downregulated and 106 genes were upregulated (Fig. S1B). These results show the clear global gene expression patterns between different treatments and controls.

The DEGs were evaluated using GO and KEGG pathway analyses to identify the genes associated with low-light tolerance in *P. bournei*. These genes involved in cells, the cell parts, membranes, the membrane parts, organelles, and the organelle parts were predominant in the cellular component category. In the biological process category, the enriched genes were involved in biological regulation, cellular component organization or biogenesis, cellular process, developmental process, localization, metabolic process, response to stimulus, and single-organism process. Similarly, genes related to binding, catalytic activity and transport activity were enriched in the molecular function category (Fig. S1C).

In order to further explore the biological functions of these DEGs, an enrichment analysis based on the KEGG database was performed. The top 20 pathways for each comparison of downregulated and upregulated genes were listed (Figs. S1D-F). For the 398 downregulated genes between T1 and CK, they were mostly enriched in the protein processing in the endoplasmic reticulum, cyanoamino acid metabolism, plant-pathogen interaction, glucosinolate biosynthesis, sulfur metabolism, and ABC transporter. 191 genes were upregulated between T1 and CK, these genes were significantly enriched in plant hormone signal transduction, monoterpenoid biosynthesis, the biosynthesis of unsaturated fatty acids, carotenoid biosynthesis, and alpha-Linolenic acid metabolism (Fig. S1D). As shown in Figure S1E, the downregulated DEGs between T2 and CK were correlated to the protein processing in the endoplasmic reticulum, cyanoamino acid metabolism, phenylpropanoid biosynthesis, anthocyanin biosynthesis, cutin-suberine, and wax biosynthesis. However, the upregulated DEGs were mostly enriched in the biosynthesis of secondary metabolites, metabolic pathways, fatty acid elongation, phenylpropanoid biosynthesis, biosynthesis of unsaturated fatty acids, isoquinoline alkaloid biosynthesis, plant hormone signal transduction, stilbenoid, diarylheptanoid, and gingerol biosynthesis (Fig. S1E). The downregulated DEGs between T2 and T1 were correlated to the protein processing in the endoplasmic reticulum, phenylpropanoid biosynthesis, cyanoamino acid metabolism, and carotenoid biosynthesis. However, the upregulated DEGs between T2 and T1 were significantly enriched in photosynthesis-antenna proteins, plant-pathogen interaction, nitrogen metabolism, and isoquinoline alkaloid biosynthesis (Fig. S1F).

### Shading affects the expression of photosystem I and II-related genes

**Fig. 2:**
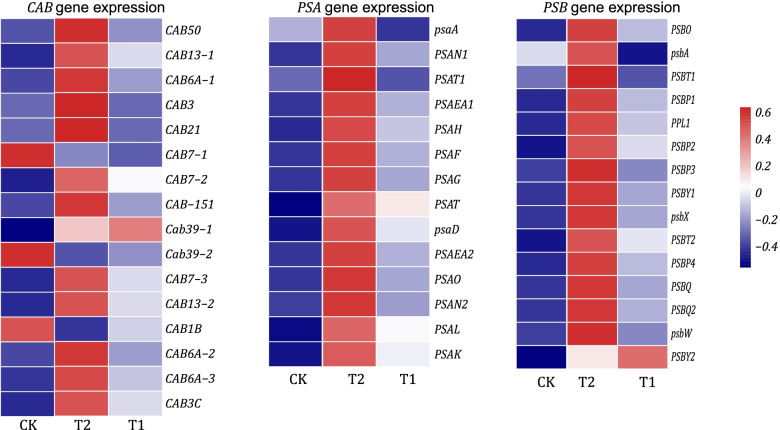
The amount of light system-related genes relative expression. The heatmap of FPKM normalized by z-score representing the expression levels of the *CAB* genes and photosystem I and II subunit genes. Most of these genes exhibited the expression patterns of T2 > T1 > CK

Photosystem I comprises more than 110 cofactors, which are significantly greater than those for photosystem II. In the present study, the transcript levels of three kinds of genes were analyzed (Fig. 2): photosystem I reaction center subunit (*PSA*), photosystem II reaction center subunit (*PSB*) and chlorophyll a-b binding (*CAB*) genes. The largest proportion of these genes was higher in T2, when compared to CK and T1, and most of which were upregulated in T2, when compared to T1 and CK. All these genes exhibited similar expression patterns. These genes were mostly expressed in T2, followed by T1 and CK. A total of 14 PSA genes were identified, and all these genes (*psaA*, *PSAT*, *PSAH*, *PSAO*, *PSAN*, *PSAF*, *PSAEA*, *PSAG*, *PSAT*, *PSAD*, *PSAL* and *PSAK*) were upregulated in T2, when compared to CK and T1. Furthermore, most of these genes had a higher expression in T1, when compared to CK, except for *psaA* and *PSAT1*. In the present study, 15 *PSB* genes were identified, and all of these (*PSBY*, *PSBP*, *PSBT*, *PSBW*, *PSBX*, *PSBQ*, *PPL*, *PSBO* and *PSBA*) had a higher expression level in T2, when compared to CK. Similarly, all these genes were upregulated in T1, when compared to CK. A total of 16 *CAB* genes were also identified. All *CAB* genes were upregulated in T2, except for *CAB1B*, *CAB7-1* and *CAB39-2*, when compared to CK. Most of these genes also had a higher expression in T1, when compared to CK.

### Shading affects the expression of structural genes and transcription factors associated with chlorophyll biosynthesis

**Fig. 3:**
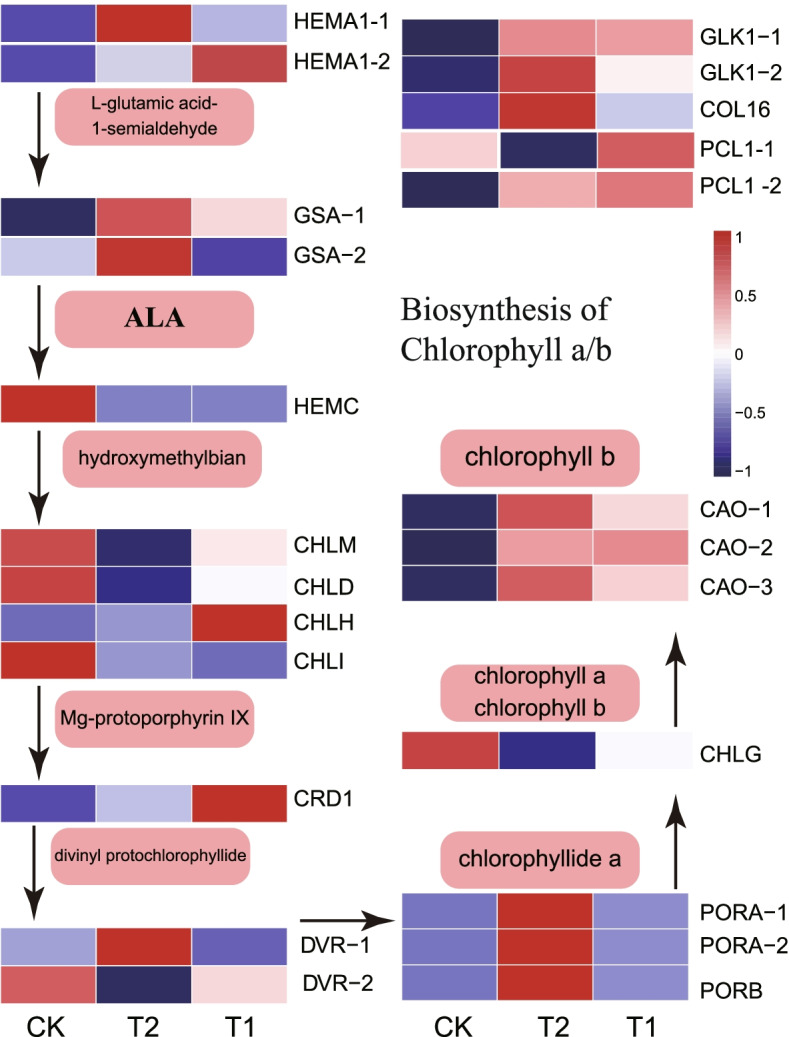
The heatmap diagrams of the relative expression levels of Chl biosynthesis-related structural genes in response to shading conditions. Heatmap showing FPKM of genes normalized by z-score. *GLK, COL16 and PCL1: T*he transcriptome factors that may be involved in the biosynthesis of Chl

The biosynthesis of chlorophyll a/b is a very complicated biological pathway, and a number of enzymes participate in the catalyzing steps. In the present study, the transcript levels of nine chlorophyll a/b biosynthesis-related structural genes were analyzed (Fig. 3). Furthermore, three kinds of transcription factors that may be involved in the biosynthesis of chlorophyll a/b were identified. The expression levels of most of those genes were high in T2 than CK. Two *HEMA1* genes were identified. *HEMA1-1* was significantly upregulated in T2, when compared to CK and T1. Among the two *GSA* genes, one *HEMC* hydroxymethylbilane synthase (porphobilinogen deaminase) gene was identified. *GSA-1* and *GSA-2* were upregulated in T2, but the *HEMC* was downregulated in T2, when compared to CK. Four *CHL* (magnesiumchelatase) genes were identified. *CHLD* and *CHLI* were downregulated in T2, when compared to CK, but the *CHLH* had a highest expression in T1. In addition, one *CHLM* gene, one *CRD1* gene, and two *DVR* genes were identified. *CHLM* and *CRD1* were downregulated in T2, when compared to CK, while *DVR-1* was significantly upregulated in T2. Three *POR* genes were identified. One *CHLG* gene and three *CAO* genes (*PORA-1*, *PORA-2* and *PORB*) were significantly upregulated in T2, when compared to T1 and CK. T2 presented with higher expression levels of *CAO-1*, *CAO-2* and *CAO-3*, when compared to CK, but the expression of *CHLG* in T2 was downregulated. In addition, five transcription factors (*GLK1-1*, *GLK1-2*, *COL16*, *PCL-1* and *PCL1-2*) were identified, and all were upregulated in T2, when compared to CK. Interestingly, it was observed that the expression levels of two genes related to chlorophyll synthesis, *ELIPs* and *EGY1*, sharply decreased after 10% light exposure, when compared to the controls, but these did not significantly change after 35% light exposure.

### Shading affects the protein processing and signal transduction

**Fig. 4:**
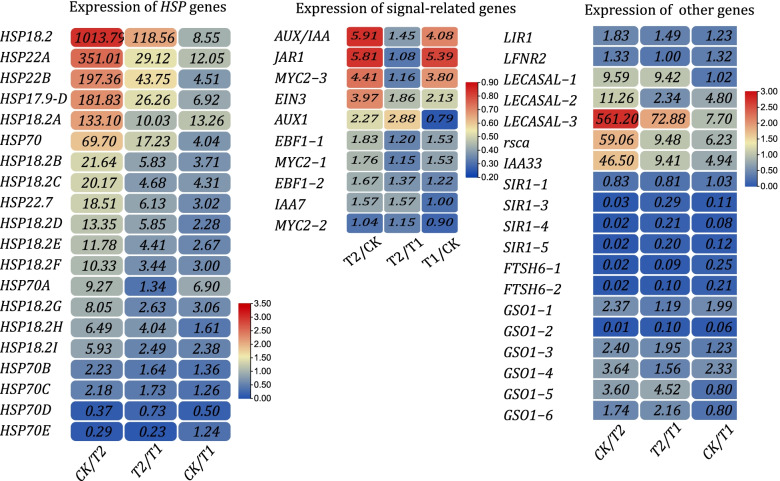
The heatmap diagrams for the expression levels of *HSP* genes, genes involved in signal transduction, and other genes are shown. The fold changes in the gene expression of these genes in T2, T1 and CK are also shown. The expression of heat shock protein family genes sharply decreased in T2, while other genes exhibited deliberate expression patterns. The numbers in the legend were the log values of fold changes in base 10

The heat shock protein (*HSP*) family plays an important role in stress resistance and plant development. In addition, *HSP* plays an important role in protein-assisted folding and error repair. To further determine the changes in the transcriptome of *P. bournei* under low light stress, the expression profile of *HSP* was determined (Fig. 4). Among the identified DEGs, *HSP18.2*, *HSP22* and *HSP22.7*, which ranged within 2—1,000 fold, were significantly upregulated in T2 compared to CK. Furthermore, eight *HSP70* genes were identified. Among these genes, six were upregulated in T2 and two genes were downregulated. It can be observed that under low light stress, the *HSP* gene expression of *P. bournei* exhibits a significant downward trend and generally conforms to the expression pattern of T2 > T1 > CK. Next, the investigators searched for genes involved in signal transduction, and identified several genes related to JA-mediated signal transduction. *MYC2*, *JAR1* and *JAZ* had an enriched expression in T2. Similarly, the expression of *IAA33* also significantly increased in T2. Finally, many genes with dramatic changes in expression under light stress were identified, such as *LECASAL*, and *RSCA* were significantly upregulated under low light stress. The expression levels of *SIR1* and *FTSH6* were significantly downregulated in T2, following the rule of CK > T1 > T2. In comparing CK and T2, the expression level of *FTSH2* was significantly downregulated, but the change was not as significant as *FTSH6* (Fig. 4).

### *MYB*, *bHLH*, *ATHB*, and other TFs may be involved in shading tolerance

**Fig. 5:**
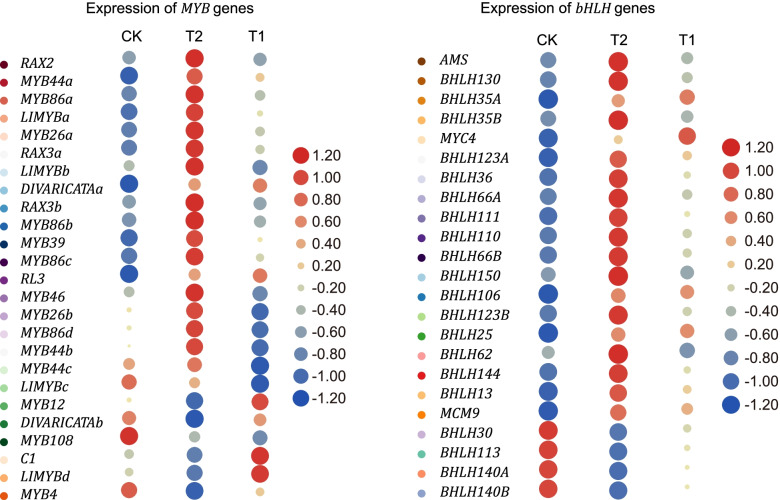
The heatmap diagrams of the relative expression levels of TFs annotated in the Chl biosynthesis in *P. bournei*. **(A)** The *MYB* TFs; **(B)** The *bHLH* TFs. Both *MYB* and *bHLH* family transcription factors had an enriched expression in T2. Heatmap showing FPKM of genes normalized by z-score. Red represents high expression level, blue represents low expression level, and the size of the circle represents the absolute value of the normalized expression level

In addition, potential transcription factors (TFs) that may be involved in shade tolerance in *P. bournei* were screened. Most of the transcription factors were derived into *MYB*, *bHLH*, *ATHB*, and other families. As shown in the figure, most of the *MYB* family transcription factors were elevated after low light, and merely five (23.8%) factors were downregulated after low light. These included *RAX2*, which is a gene that may be involved in photo-reactivity and the upregulated expression. Similarly, most *bHLH* family transcription factors also appeared with an enriched expression in low light condition. The results revealed that a total of 24 *bHLH* TFs were significantly upregulated in T2, when compared to CK, and merely four of these were downregulated (Fig. 5). The upregulated expression pattern of transcription factors and the downregulated expression of *HSP* genes were in sharp contrast.

### qRT-PCR validation of differentially expressed genes

**Fig. 6:**
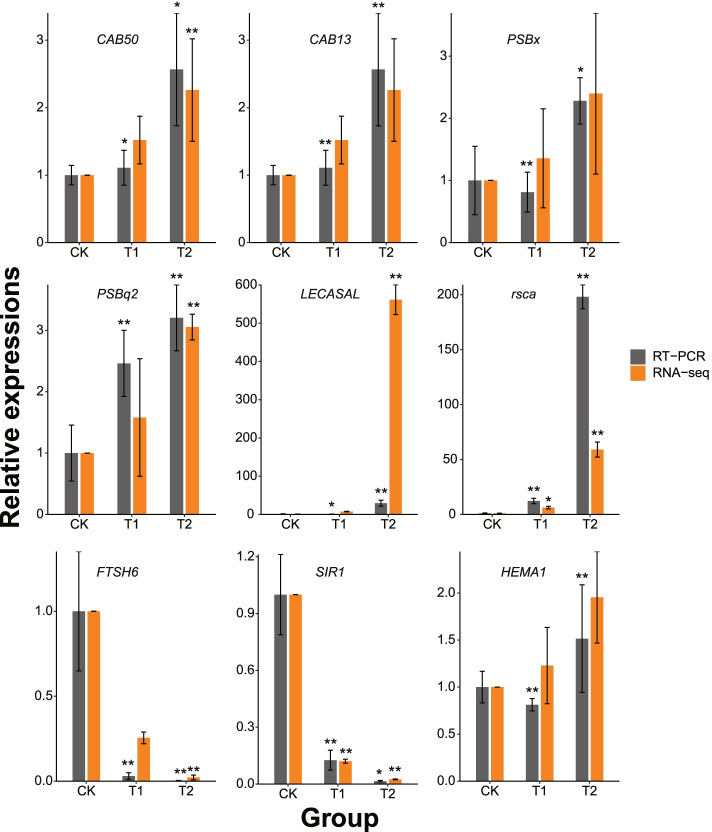
The RT-PCR results confirm the transcriptome expression of the RNA sequencing. Nine genes related to light response were selected for verification. The data was standardized as the *ACT2* expression value. The bars denote standard deviation values (*n* = 3). There was a significant difference between the control group and shading treatment group (T-test, * *P* < 0.05, ** *P* < 0.01)

Among all the identified DEGs, genes correlated to the light-harvesting complex, porphyrin, chlorophyll metabolism and chlorophyll synthesis were closely correlated to the changes in leaf color observed during shading. Nine of these were selected for the quantitative real-time PCR (qRT-PCR) analysis. In the 35% light exposure group, the expression patterns of seven genes detected by qRT-PCR were similar to those observed in the DEG data, while the *PSBx* and *HEMA1* genes exhibited slightly different expression patterns, when compared to the DEGs. In the 10% light exposure group, all genes exhibited the same expression pattern as the DEGs (Fig. 6). In general, the qRT-PCR data was consistent with the Illumina sequencing results, indicating that the RNA sequence data is reliable.

## Discussion

*P. bournei* is distributed in southeastern and southwestern of China, and is a national secondary protected plant. Although low-light stress has an adverse effect on plant growth and development. *P. bournei* has strong adaptability to low light, and it can grow properly under shaded conditions. Numerous studies have shown considerable underlying mechanisms of the shade tolerance of plants [20, 21]. Shade-tolerant plants have lush foliage, large leaf areas, no stratum corneum or very thin stratum corneum, less stomata, and chloroplasts [22, 23]. The leaf area of *P. bournei* seedlings is T2 > T1 > CK, which indicates that the stronger the light, the smaller the leaf area. The chlorophyll content of plants with strong negative shade tolerance is higher than that of plants with weak shade tolerance. Plants with stronger negative shade tolerance have higher chlorophyll b and lower chlorophyll a/b ratio [24]. These index characteristics are consistent with the high chlorophyll b and low chlorophyll a/b value in T2. Under the low light conditions, plants with lower chlorophyll a/b value and higher chlorophyll content also had higher photosynthetic activity [25, 26]. The light response curve characteristics and daily changes in T1 were higher than those in T2 and CK. At the same time, T2 maintains higher photosynthetic activity. Under suitable light conditions, Pn would also be higher. In the present study, the morphological and transcriptional changes of *P. bournei* under different shading conditions were demonstrated to reveal the molecular mechanism of *P. bournei* resistance to shading [27]. Under 35% shading conditions (T1), the leaves were emerald green with high chlorophyll content. Under 10% shading treatment (T2), the leaf color turned dark green, with higher chlorophyll content, and the ratio of chlorophyll a/b also significantly decreased, while the leaf color of *P. bournei* that grew under full sunlight was yellow-green. These changes indicated that the content of Chl b and total chlorophyll increased with the decrease in light intensity, and that the value of Chl a/b decreased with the increase in shade tolerance. Furthermore, the absorption spectrum of chlorophyll b in blue-violet light is wider than that of chlorophyll a. Under shading conditions, the proportion of astigmatism dominated by blue-violet light increases, and the increase in chlorophyll b content would be more conducive to the absorption of blue-violet light by *P. bournei*. The significant decrease in Chl a/b value also indicates that chlorophyll a accelerates the conversion to chlorophyll b. The present transcriptome data also revealed the significant upregulation of the CAO gene expression in T2. Furthermore, the current data revealed that the content of carotenoids in T2 and T1 also significantly increased. Previous studies have shown that carotenoids in green leaves ensure effective photosynthesis, remove various reactive oxygen species, and protect chlorophyll from photooxidation [28].

The present study revealed that a number of related genes in photosystem I and photosystem II are upregulated in T2, and exhibit an expression pattern of T2 > T1 > CK. In the present expression profiling data, the expression levels of *psaN*, *psaL*, *psaK*, *psaH*, *psaD*, *psaEA* and *psaO* were significantly upregulated in T2, and the expression levels of the *CAB6A* and *CAB151* genes were significantly enriched in T2 and lowest in CK. The catalytic center within the PSII core complex consists of four of the largest intrinsic subunits: *PsbA* (D1), *PsbB* (CP47), *PsbC* (CP43) and *PsbD* (D2). The other membrane intrinsic small subunits include *PsbE*, *PsbF*, *PsbH-M*, *PsbTc*, *PsbW*, *PsbX* and *PsbZ*. The external antenna system consists of a monomeric pigment-protein complex, and the photosystem II core is in direct contact with the trimer LHCII, which acts as a heterotrimer that comprises of *Lhcb1*, *Lhcb2* and *Lhcb3* [29]. Similarly, the present study revealed that the *psbO*, *psbY*, *psbX*, *psbP* and *psbW* genes were enriched in T2, and that the expression levels of *CAB50*, *CAB13* and *CAB3C* also significantly increased in T2, but were the lowest in CK. Particularly, chlorophyll forms an effective chlorophyll protein complex by binding to *PSA*, the *PSB* subunit, and the *LHC* protein. The upregulated expression of these genes increases the effective content of chlorophyll in leaves, and increases the efficiency of light-harvesting molecules in the leaves of *P. bournei* under low light, thereby increasing the effect of photosynthesis. Therefore, negative-tolerant plants can increase the expression level of photosystem-related genes in low light as a corresponding mechanism of low light stress. The upregulation of the expression of these genes increased the effective content of chlorophyll in leaves, and increased the efficiency of the leaf light trapping molecules of *P. bournei* under low light, thereby improving the effect of photosynthesis. Therefore, negative-tolerant plants can increase the expression level of photosystem-related genes under low light, as a corresponding mechanism of low light stress.

The transcriptome analysis provides genes that respond to low light stress in the chlorophyll biosynthesis pathway, which may be correlated to the shade tolerance of *P. bournei*. The biosynthesis of chlorophyll is a complex process that involves a number of structural genes and transcription factors [30]. It was found that in T2, *HEMA1*, *GSA*, *PORA*, *PORB*, *CAO*, and other structural genes were upregulated. Among these, *HEMA1* was the first step of chlorophyll biosynthesis, while *CAO* accelerated the conversion of chlorophyll a to chlorophyll b. *PORA* and *PORB* were responsible for the synthesis of chlorophyll b. Obviously, the upregulation of *CAO* led to the conversion of chlorophyll a to chlorophyll b. The decrease in chlorophyll a/b value enhanced the absorption of blue-violet light in *P. bournei* under low light [31]. The *POR* enrichment expression increased the chlorophyll content of *P. bournei* under the shade when compared to previous studies. Consistently, increasing the chlorophyll content as an adaptive mechanism for shade-tolerant species can promote the maximization of light capture. The present expression data revealed that *GLK1* had the highest expression in T2, followed by T1, and this was the lowest in CK. This may indicate that *GLK1* is also involved in the biosynthesis of chlorophyll in *P. bournei*, the expression of the photosystem subunit protein, and the antenna protein. In particular, the investigators also identified another transcription factor, *COL16*. Previous studies have shown that *COL16* was involved in the chlorophyll accumulation in morning glory, the expression of *phCOL16* increased in the corolla chlorophyll level and increased the gene expression involved in chlorophyll biosynthesis, and positively regulates the biosynthesis of chlorophyll [31]. The present study revealed that the expression of *COL16* sharply increased in T2, and increased to some extent in T1, but was the lowest in CK. This indicates that *COL16* also plays a role in the regulation of chlorophyll biosynthesis. The expression of *COL16* promoted the accumulation of chlorophyll in *P. bournei*, inducing the leaves to be darker. However, in the CK, the expression of *COL16* was the lowest, and the leaves were yellow-green, which is consistent with previous studies [32].

Jasmonic acid (JA) with its derivative, methyl jasmonic acid, is an important signaling molecule for plants against biotic or abiotic stress due to environmental stress. This activates the activity of a number of correlated transcription factors to regulate plant defense responses [33, 34]. *MYC* transcription factors are the core transcription factors in the response pathway of JA hormones in plants. *MYC* transcription factors have a variety of regulatory functions, and are widely present in plants and animals [35]. Among the discovered plant *MYC* transcription factors, *MYC2* is mostly used in in-depth studies. At present, the *MYC2* transcription factor found in the model plant Arabidopsis plays a regulatory role by forming the *COI1*/*JAZs*/*MYC2* complex. This participates in the signal transduction process of JA, ABA, and other hormones [36, 37]. In the present study, it was found that *JAR1*, which is an important gene involved in JA metabolism, and *MYC2*, *JAZ1*, *JAZ2*, *JAZ3* and *JAZ12*, which are involved in JA signal transduction, were significantly upregulated in T2. The association of SAS with the suppression of the JA- and SA-mediated responses involved in plant defense against insect/pathogen and disease resistance was well-characterized in *A. thaliana*. JA-mediated defenses are repressed by low light intensity in shade-intolerant, but enhanced shade-tolerant, wild species [9]. The present results indicate that the JA-mediated defense response is significantly enhanced under low light stress, showing that that JA signaling pathway plays an important role in the low light adaptation of *P. bournei*.

For the identified DEGs, the expression levels of most *HSP* genes were significantly reduced in T2, while most of the *bHLH* and *MYB* transcription factors were upregulated in T2. *HSPs*, which are evolutionary conserved and found in all living organisms, are responsible for proper protein (re)folding, assembly, translocation, and stabilization, as well as protein protection and degradation. After various stress conditions [38], it was found that *HSP18.2*, *HSP22* and most of the *HSP70* were downregulated in T2, while two *HSP70* were upregulated in T2. Combined with the biological function of the *HSP* gene, the metabolism of the protein in *P. bournei* slowly down under weak light stress. *HSP* increases its expression under strong light, and has photoprotective effects in various light intensities and organisms [39, 40]. This is similar to the present results. *P. bournei* inhibited the unnecessary defense mechanism by regulating the expression of *HSP* under low light. This is of great significance for the growth of *P. bournei*. Studies have shown that the *bHLH* transcription factor also regulates plant response of environment changes, such as controlling the light response and interacting with components of the circadian clock [41, 42]. In addition, *bHLH*-like transcription factors may also play a role in the tolerance of *P. bournei*. For instance, the expression level of *RAX2*/*MYB38* from the *MYB* transcription factor family was significantly upregulated in T2. Furthermore, previous studies have revealed that *RAX2* may play a role in the response of plants in blue light [43]. Under low light conditions, the proportion of blue light would increase. Hence, *RAX2* may play the same role in *P. bournei*. Furthermore, various *MYB* transcription factors have been identified to upregulate its expression in low light, confers greater resistance to *P. bournei*.

Furthermore, it was found that the expression levels of *rsca*, *IAA33* and *LECASAL* significantly increased in T2, but maintained extremely low expression levels in CK and T1, suggesting that these genes are involved in the low light response of *P. bournei*. *Rsca* is a chitinase, and is considered to be involved in the defense response to the fungus cell wall macromolecule catabolic process [44, 45]. Under low light stress, the expression of *rsca* in southern Fujian sharply increased, which may indicate that *rsca* also plays an important role in plant low light adaptation. *IAA33* is a member of the Aux/IAA family, and is a short-lived transcription factor that can act as an inhibitor for the early auxin A gene at low auxin concentrations [46]. The high expression of *IAA33* inhibits the effective activity of auxin, indicating that *P. bournei* regulates the growth of different parts of the plant by regulating the expression of the *IAA33* gene in low light. In addition, the high expression of *IAA33* may also inhibit protein biosynthesis. In terms of growth height, T1 > T2 > CK is likely to be closely correlated to the high expression of *IAA33* under low light conditions. *LECASAL*, which is a mannose-specific lectin, is associated with the cell–cell adhesion defense response to insects [47]. The present study provides a new perspective for studying the function of *lecasal*. *Lecasal* is induced by low light in *P. bournei*, and its expression sharply increases, which may indicate that *lecasal* is also involved in the low light adaptation of *P. bournei*.

## Materials and methods

### Materials and *P*. *bournei* growth condition

*P. bournei* seeds were collected from Rongjiang County in Guizhou Province in 2015, China, and then cultivated in the nursery of Guizhou University. The authority responsible for the *P. bournei* resources is the Rongjiang County Forestry Bureau in Guizhou Province, China, who provided permission to collect the seeds of *P. bournei*. The formal identification of the plant material was undertaken by Prof. Mingtai An (Guizhou University). The experiment was carried out in the nursery of the Guizhou University (Huaxi District, Guiyang City, Guizhou Province, longitude 104°34', latitude 26°34', altitude 1,159 m). Two-year-old *P. bournei* container seedlings with the same growth status were used as experimental materials and planted in 17.6 × 14.5 cm flower pots. The potting soil used was loam soil: the humus was prepared at a ratio of 1:1. Three gradient treatments were set, 100% (CK), 35% light intensity (T1), and 10% light intensity (T2). The different light intensities measured by Spectrum Technologies (ICN, San Diego, CA, USA). Sunshade nets (the height was 1.5 m, the width was 1.4 m, and the length was 3 m) with different light transmittances were used for shading. The distance between the north and south sunshade nets was equal to that of the ground. A total of 90 plants were divided into three replicates, with 10 seedlings per replicate. The physiological, morphological and transcriptome sequencing materials were selected according to the changes in chlorophyll content as leaves shaded for 30 days.

### Determination of morphological and physiological indicators

The morphological and physiological indicators were measured after 30 days of shading treatment, three plants per treatment, and one leaf of *P. bournei* seedlings per plant. The photosynthetic determine used the Li-6400XT portable photosynthesis analyzer (Li-COR, Lincoln, NE, USA) at 1,200 μmol m^−2^ s^−1^ as the induced light intensity. Photosynthetic light response curves were tested with light intensities ranging from 1000 to 0 μmol m^−2^ s^−1^. The respiration rate in dark (Rd) and light-saturated photosynthetic rate (Pmax) were obtained during the measurements of light response curves which were fitted with the supporting software of Photosynthesis. The Gs, Tr and intercelluar CO_2_ concentration (Ci) were calculated under saturated light. For each treatment, one seedling was selected, dried until green at 105 °C for half an hour, and continued to dry at 65 °C for 48 h. Then, the weight and dry weight were determined to calculate the biomass.

Statistical analysis was performed using One-Way analysis of variance (ANOVA) by the means and standard errors (means ± SE) at least three replicates with SPSS 18.0 (Chicago, IL, Armonk, NY, USA).

### RNA extraction and Illumina sequencing

Three plants were collected from each sample set, and 3–6 leaves from each plant were quickly placed in a container with liquid nitrogen and sent to Guangzhou GENE DENOVO Biotechnology Co., Ltd. (Guangzhou, China) for RNA extraction and sequencing. Total RNA from plant leaves extracted by TRIzol reagent, and then enrich the mRNA through oligdT. The sequencing libraries were generated using NEBNext® Ultra™ RNA Library Prep Kit for Illumina® (NEB, Ipswich, MA, USA), according to the manufacturer’s recommendations. The library quality was assessed using the Agilent Bioanalyzer 2100 system, then sequenced using the Novaseq 6000 platform (Illumina, San Diego, CA, USA).

### De novo assembly, expression, DEGs and enrichment analysis of *P*. *bournei* transcriptome

The high-quality clean reads were obtained from the sequencing machines after filtering, following strict rules. Then, the sequence with rRNA reads removed were used for the de novo assembly by Trinity (reference). The gene expression level was quantified using the HTseq software [48], and normalized using the FPKM (Fragments Per Kilobase of transcript per Million mapped reads) method. DEGs across the treatments and controls were identified using the edgeR package in R (http://www.r-project.org/). The fold change (FC) and false discovery rate (FDR) were used to screen the significant DEGs, and the genes with FDR < 0.05 and |log2FC|> 1 was identified as differentially expressed genes (DEGs) [49]. Then, these DEGs were subjected to functional enrichment analysis. After drawing the Venn diagram using the VennDiagram and UpSetR package of R, the differential genes data for CK, T1 and T2 were analyzed.

The clusterProfiler software package was used for the Gene Ontology (GO) enrichment analysis based on the GO database (http://www.geneontology.org/). Pathway enrichment analysis was performed using the Kyoto Encyclopedia of Genes and Genomes (KEGG, www.kegg.jp/kegg/kegg1.html) [50] database by R software. At the same time, the *P*-value was obtained by the hypergeometric test and corrected, correction *P*-value ≤ 0.05 were identified to significantly enriched GO terms or pathway.

### The qRT-PCR validation of target genes

All components were configured for the qTOWER2.2 Real-time PCR System (Analytik Jena AG, Jena, Germany) and centrifuged at 6,000 rpm for 30 s at 4 °C using a PCR plate centrifuge. Then, samples were placed into the quantitative PCR instrument and amplified according to the above procedure. The amplification steps were as follows, the fluorescence quantitative PCR program and system: (a) Step 1, 95℃ for three minutes; (b) Step 2, 95℃ for 10 s; (c) Step 3, 58℃ for 30 s + plate read; (d) Step 4, Go to Step 2, 39 cycles; (e) Step 5, melt curve analysis (60–95℃, + 1℃/cycle, holding time = four seconds).

The nine samples were separated, and three duplicate wells were set up. Then, the relative expression of the target gene in each sample was automatically calculated using the instrument software qPCRsoft 3.2 through the Pfaffl method. Then, ACT2 was used as the internal reference, and the CK group was used as the control group to estimate the relative expression.

## Supplementary Information

**Additional file 1: Figure S1. **The differential analysis of the gene expression among treatments. **A** The comparison of the number of upregulated and downregulated genes among CK, T1 and T2. **B** The Wayne graph statistics for the differential genes is shown. **C** The GO pathway enrichment analysis between the controls and treatments. **D-F** The KEGG pathway enrichment analysis between the controls and treatments.

## Data Availability

The raw sequencing data has been uploaded to the SRA database and obtained through the accession number SRR13842622 to SRR13842630.

## Abbreviations

Chl: Chlorophyll
DEG: Differentially expressed genes
RNA-seq: RNA sequencing
LSP: Light saturation point
LCP: Light compensation point
P_max_: Maximum photosynthetic rate
Rd: Respiration rate in dark
Tr: Transpiration rate
Gs: Stomatal conductance
Ci: Intercelluar CO_2_ concentration
GO: Gene Ontology
KEGG: Kyoto Encyclopedia of Genes and Genomes
HSP: Heat shock protein
CAB: Chlorophyll a-b binding
HEMA: Glutamyl-tRNA reductase
GSA: Glutamate-1-semialdehyde2,1-aminomutase
HEMC: Hydroxymethylbilane synthase
CHLM: Magnesium-protoporphyrin IX methyltransferase
CHLD: Magnesium chelatase subunit L
CHLH: Magnesium chelatase subunit H
CHLI: Magnesium chelatase subunit I
CHLG: Chlorophyll /bacteriochlorophyll a synthase
CRD: Chloroplast Relocation Defective
DVR: 3,8-Divinylprotochlorophyllidea 8-vinyl reductase
POR: NADPH-protochlorophyllide oxidoreductase
CAO: Chlorophyllide a oxygenase
TF: Transcription factors
MYB: V-myb avian myeloblastosis viral oncogene homolog
bHLH: Basic helix-loop-helix
PSA: Photosystem I reaction center subunit
PSB: Photosystem II reaction center subunit
qRT-PCR: Quantitative real-time PCR
