# Supplementary figures and images for: Transcriptome analysis reveals the accelerated expression of genes related to photosynthesis and chlorophyll biosynthesis contribution to shade-tolerant in Phoebe bournei
Source: BMC Plant Biol. 2022 Jun 1;22:268. doi: 10.1186/s12870-022-03657-y (PMC9158164; doi:10.1186/s12870-022-03657-y)

DiffExp Gene Statistics

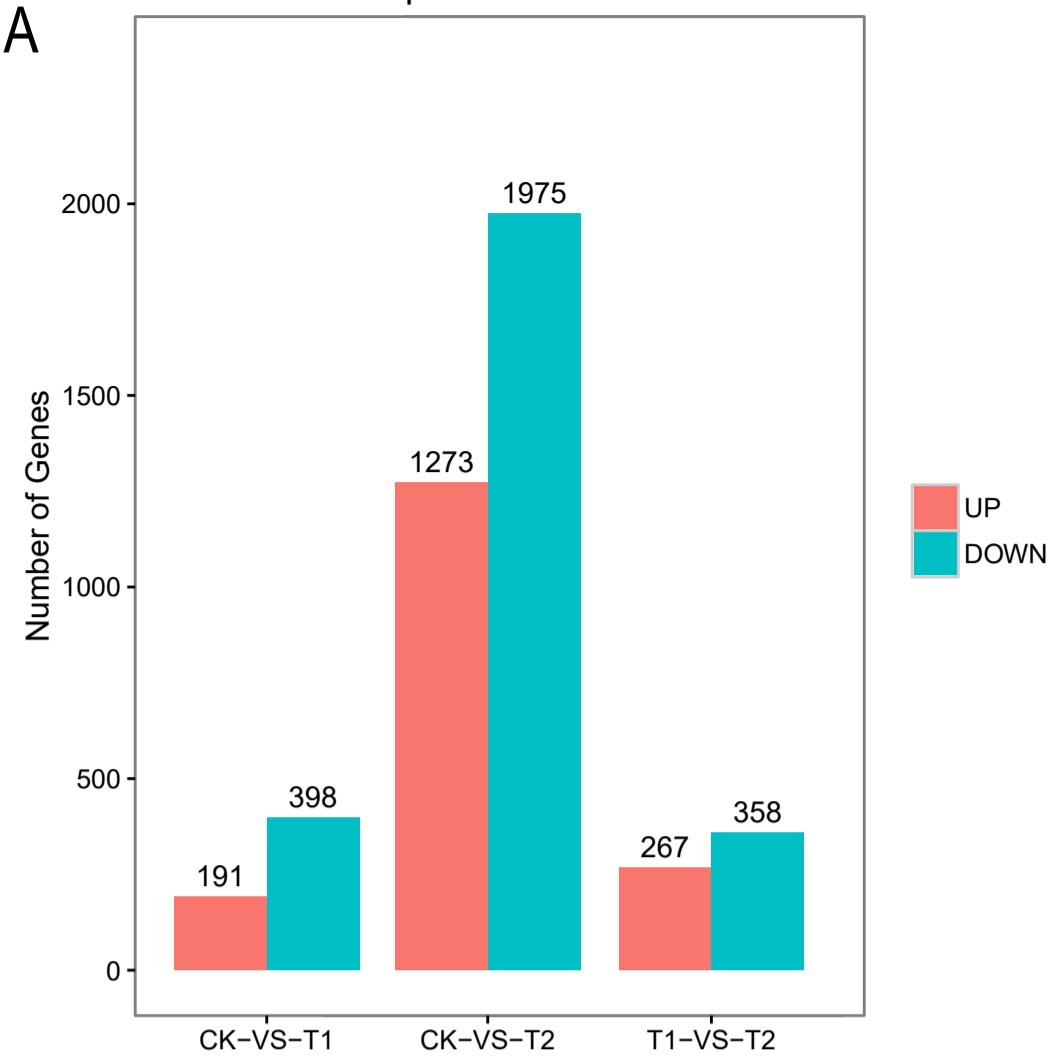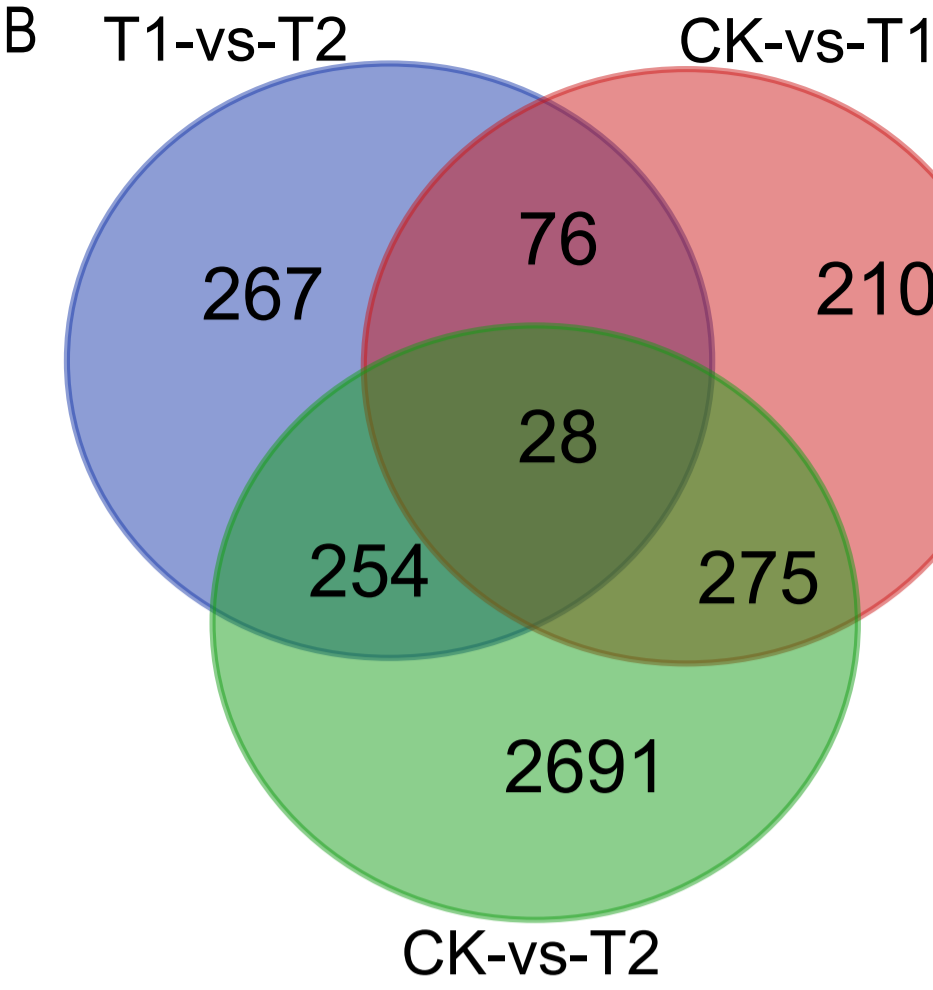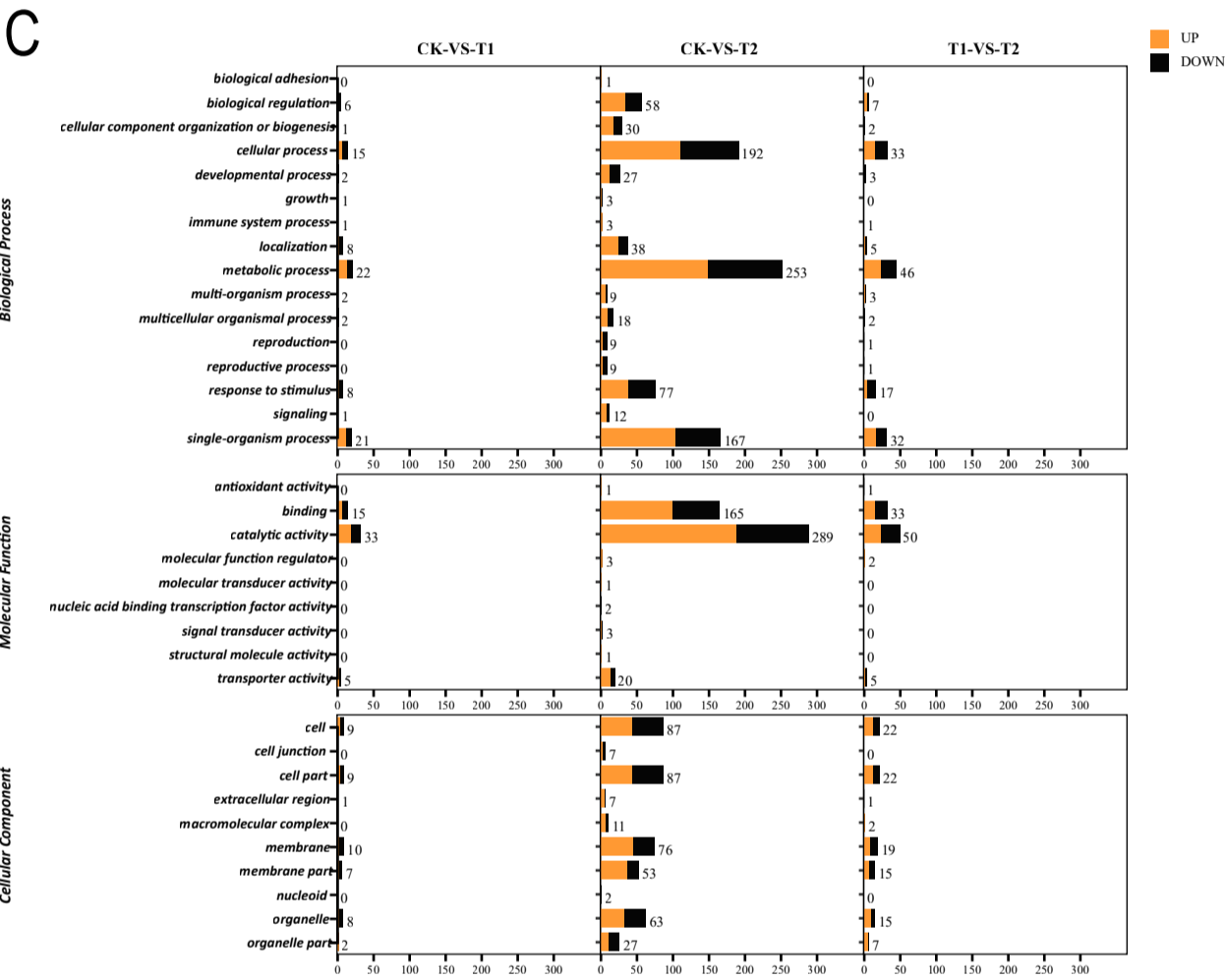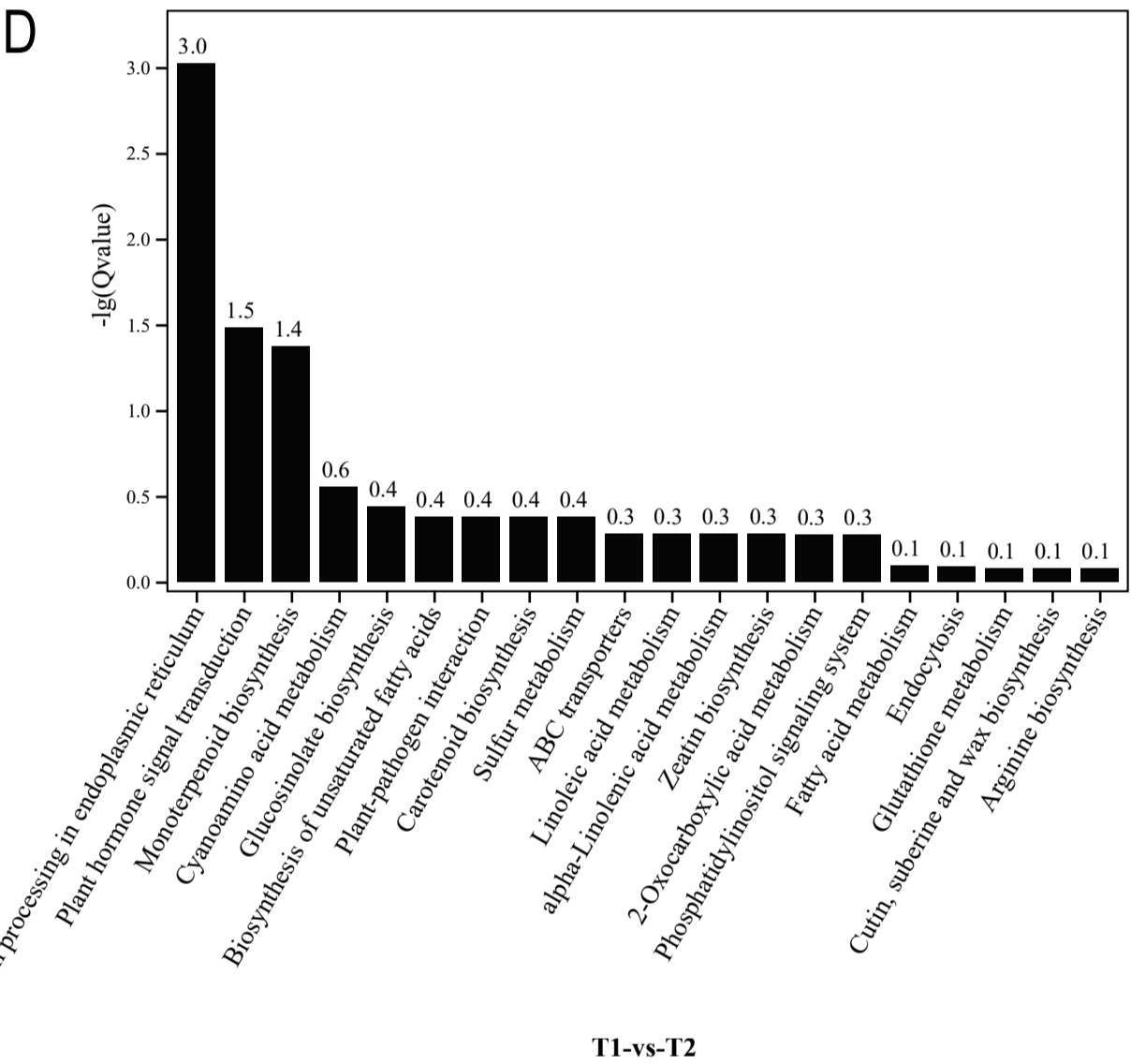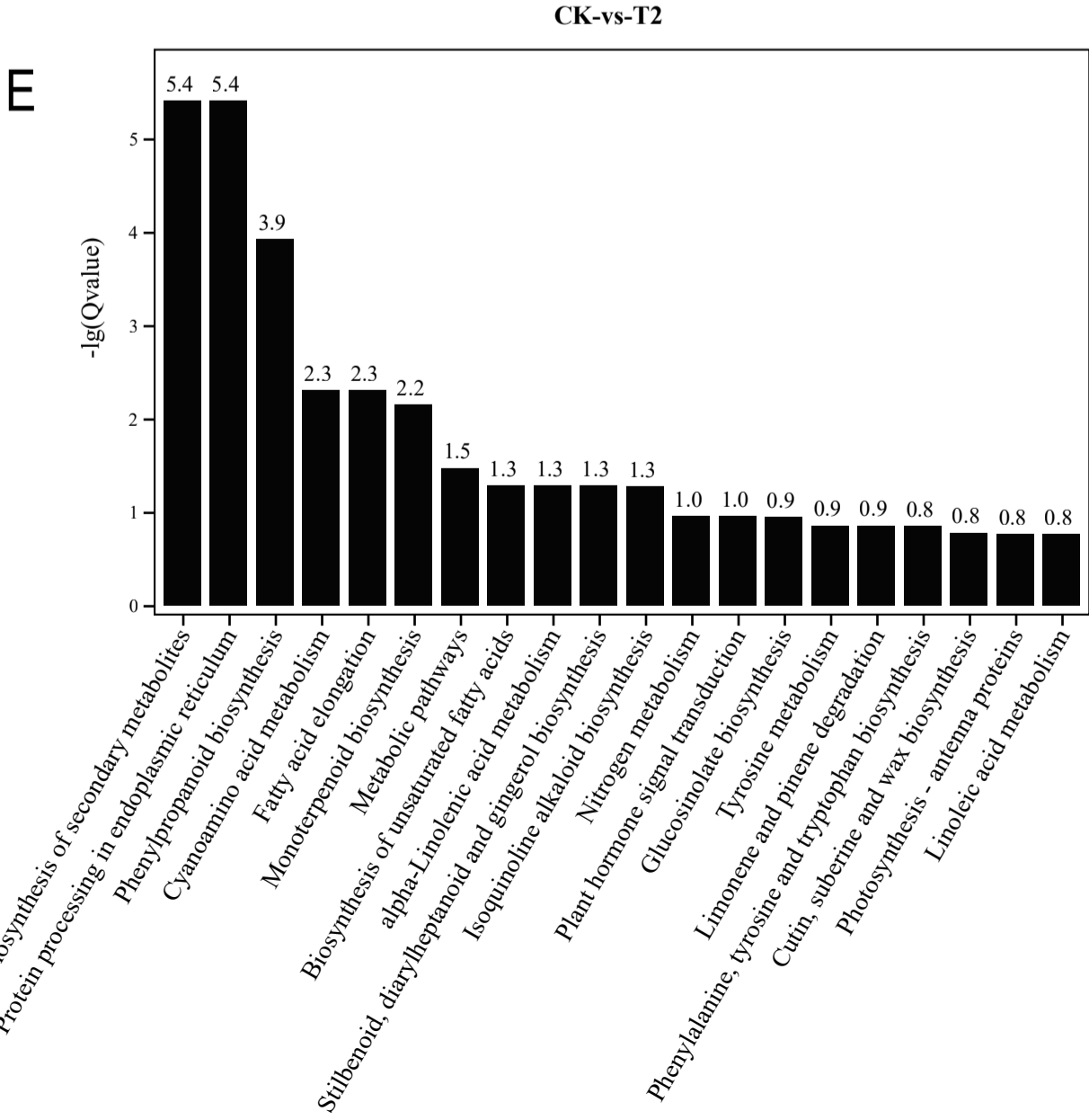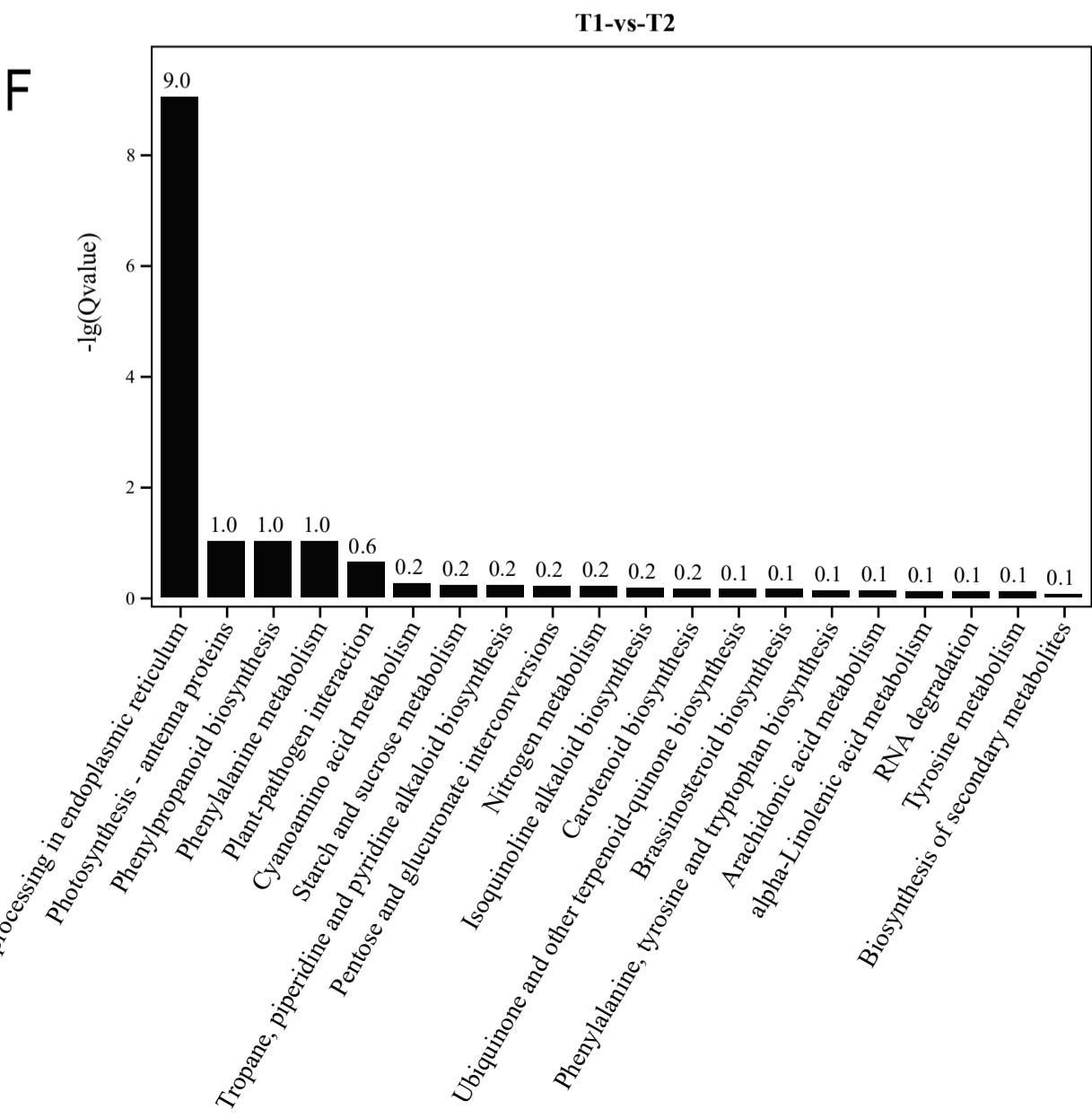

Supplement: Supplementary file 1 — Additional file 1: Figure S1. The differential analysis of the gene expression among treatments. A The comparison of the number of upregulated and downregulated genes among CK, T1 and T2. B The Wayne graph statistics for the differential genes is shown. C The GO pathway enrichment analysis between the controls and treatments. D-F The KEGG pathway enrichment analysis between the controls and treatments. [file 12870_2022_3657_MOESM1_ESM.pdf]
